# Inhibition of Myocardial Cell Apoptosis Is Important Mechanism for Ginsenoside in the Limitation of Myocardial Ischemia/Reperfusion Injury

**DOI:** 10.3389/fphar.2022.806216

**Published:** 2022-03-01

**Authors:** Zhihan Chen, Jingping Wu, Sijing Li, Caijiao Liu, Yulan Ren

**Affiliations:** ^1^ School of Acupuncture Moxibustion and Tuina, Chengdu University of Traditional Chinese Medicine, Chengdu, China; ^2^ Department of Medical Cosmetology, Affiliated Hospital of Chengdu University of Traditional Chinese Medicine, Chengdu, China; ^3^ School of Chinese Classics, Chengdu University of Traditional Chinese Medicine, Chengdu, China

**Keywords:** ginsenosides, apoptosis, myocardial ischemia/reperfusion injury, *Panax ginseng*, review

## Abstract

Ischemic heart disease has a high mortality, and the recommended therapy is reperfusion. Nevertheless, the restoration of blood flow to ischemic tissue leads to further damage, namely, myocardial ischemia/reperfusion injury (MIRI). Apoptosis is an essential pathogenic factor in MIRI, and ginsenosides are effective in inhibiting apoptosis and alleviating MIRI. Here, we reviewed published studies on the anti-apoptotic effects of ginsenosides and their mechanisms of action in improving MIRI. Each ginsenoside can regulate multiple pathways to protect the myocardium. Overall, the involved apoptotic pathways include the death receptor signaling pathway, mitochondria signaling pathway, PI3K/Akt signaling pathway, NF-κB signaling pathway, and MAPK signaling pathway. Ginsenosides, with diverse chemical structures, regulate different apoptotic pathways to relieve MIRI. Summarizing the effects and mechanisms of ginsenosides contributes to further mechanism research studies and structure–function relationship research studies, which can help the development of new drugs. Therefore, we expect that this review will highlight the importance of ginsenosides in improving MIRI *via* anti-apoptosis and provide references and suggestions for further research in this field.

## Introduction

Ischemic heart disease (IHD) is characterized by insufficient blood flow to the cardiac tissue (Querio et al., 2021). In 2019, “The top 10 causes of death” presented that IHD was the world’s biggest killer, responsible for 16% of total deaths worldwide (WHO, 2020). Myocardial blood flow blockage causes inflammatory reactions, energy metabolism disorders, micrangium damage, oxidative stress, calcium overload, and arrhythmia (Hao et al., 2021; Vilela and Fontes-Carvalho, 2021). Reperfusion therapy is the standard therapy for IHD; nevertheless, the restoration of blood flow to ischemic areas aggravates myocardial damage, namely, myocardial ischemia/reperfusion injury (MIRI) (Neri et al., 2017; Li et al., 2019). Evidence indicated that the death rate of acute myocardial infarction (AMI) patients treated with optimal reperfusion therapy was approximately 7% (Hausenloy and Yellon, 2016). MIRI involves multiple regulatory mechanisms, such as cell death, oxidative stress response, and mitochondrial dysfunction (Neri et al., 2015; Dong et al., 2019; Bugger and Pfeil, 2020). Apoptosis, which is a programmed cell death, is the critical factor in the development of MIRI (Charununtakorn et al., 2016). Apoptosis causes myocardial infarction, damages cardiac systolic/diastolic dysfunction and electrophysiological performance (Li CY. et al., 2020; Cai et al., 2020), and even leads to irreversible damage (Liu et al., 2015). Previous studies indicated that inhibiting apoptotic pathways could effectively alleviate MIRI (Zhai et al., 2017; Cai et al., 2020; Liao et al., 2021). Regulating apoptosis is a promising therapeutic strategy.

Ginsenosides are triterpenoid saponins, which are deemed as the main bioactive components of *Panax ginseng* (Sabouri-Rad et al., 2017). *Panax* means “all healing” in Greek (Im and Nah, 2013), and *Panax ginseng* has effects in improving arrhythmia, decreasing the myocardial ischemic area, suppressing oxidative stress response, enhancing immune regulation, and inhibiting apoptosis (Sun et al., 2016; Wang H. et al., 2021). Ginsenosides have positive effects on MIRI via regulating oxidative stress, inflammation, calcium overload, and cell deaths (Fan et al., 2020; Wang et al., 2020). Previous evidence indicated that ginsenosides could improve myocardial cell (MC) apoptosis to promote cardiac functions and reduce infarct size in MIRI (Zhang et al., 2016; Li CY. et al., 2020). Ginsenosides are classified into three types: protopanaxadiol (PPD) type, oleanolic acid type, and protopanaxatriol (PPT) type (Sun et al., 2016). Ginsenosides inhibit MC apoptosis via different apoptotic pathways, owing to their distinct chemical structures (Kim et al., 2015).

In this review, PubMed, Embase, Web of Science, and China National Knowledge Infrastructure (CNKI) were searched from inception to 21 September 2021 by using the following terms: ginsenoside, myocardial reperfusion injury, etc. This research included and reviewed research studies addressing the anti-apoptosis effects of ginsenosides on MIRI to provide references and suggestions for further research in this field.

## Myocardial Cell Apoptosis in Myocardial Ischemia/Reperfusion Injury

Myocardial ischemia (MI) is a complex pathological condition resulting from initial restriction of blood supply to the heart (Korshunova et al., 2021), which causes tissue hypoxia (Eltzschig and Eckle, 2011) and impediment of re-synthesis of energy sources (e.g., ATP) (Gunata and Parlakpinar, 2021). The lack of ATP reduces the activity of sodium–potassium pumps on the membrane, leading to calcium overload (Zhang et al., 2020). Calcium overload induces arrhythmias (Tribulova et al., 2016; Sugiyama et al., 2021), mitochondrial dysfunction (Tian and Zhang, 2021), and MC apoptosis (Gao et al., 2021). The recommended therapy of MI is reperfusion, namely, restoration of blood flow to ischemic areas (Jneid et al., 2017). Nevertheless, it also causes further myocardial damage (Virani et al., 2020; Ren et al., 2021). MIRI is characterized by metabolic disturbance, cardiac dysfunction, inflammatory reaction, and cell death (apoptosis, autophagy, necrocytosis, pyroptosis, ferroptosis) (Moens et al., 2005; Eltzschig and Eckle, 2011; Hwang et al., 2021; Lv et al., 2021; Peng et al., 2021).

**FIGURE 1:**
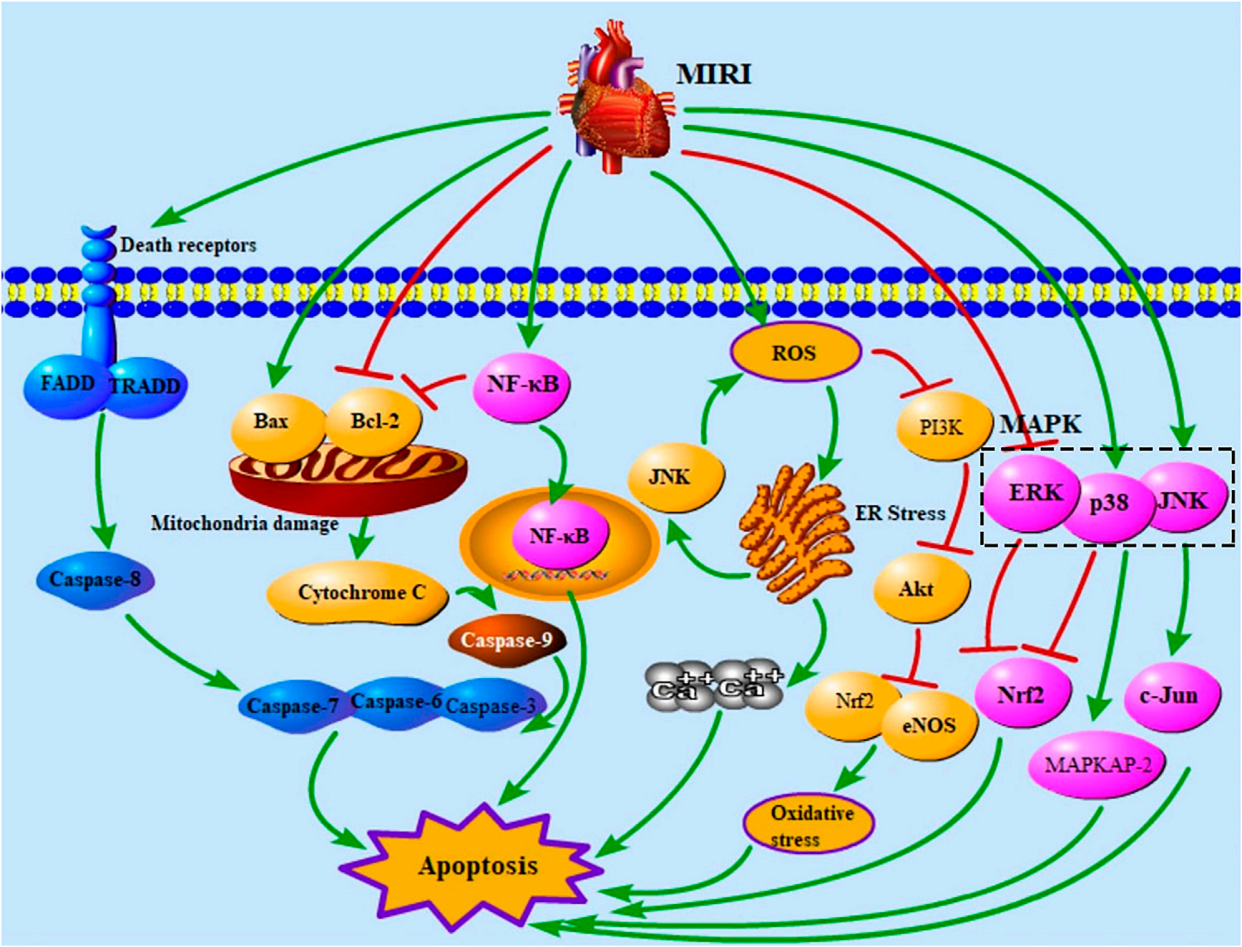
Apoptotic pathways in MIRI. MIRI, myocardial ischemia/reperfusion injury; FADD, Fas-associated death domain protein; TRADD, TNFR1-associated death domain protein; NF-κB, nuclear factor of kappaB; ROS, reactive oxygen species; ER, endoplasmic reticulum; PI3K, phosphatidylinositol-3-kinase; MAPK, mitogen-activated protein kinase; ERK, extracellular signal–regulated kinase; Nrf2, nuclear factor E2–related factor 2; eNOS, endothelial nitric oxide synthase; c-Jun, c-Jun N-terminal kinase; MAPKAP-2, MAPK-activated protein kinase-2.

Cell death occurs widely during pathological processes in multiple diseases and is one of the leading causes of death (Wang F. et al., 2021). Apoptosis is a type of programmed cell death, characterized by cell shrinkage, chromatin condensation, and nuclear shrinkage (Hotchkiss et al., 2009). Apoptosis pathways include the death receptor apoptosis pathway, mitochondria apoptosis pathway, endoplasmic reticulum (ER) pathway, PI3K/Akt signaling pathway, NF-κB signaling pathway, and mitogen-activated protein kinase (MAPK) signaling pathway (Hotchkiss et al., 2009; Lai et al., 2021; Zhu and Zhou, 2021). Death receptors are activated by their ligands, namely, FasL, TNF-α, and TRAIL (Zhang et al., 2019a). Death-inducing signaling complex formed by receptors and ligands can activate caspase-8, and activated caspase-8 further up-regulates caspase-3, caspase-6, and caspase-7, resulting in apoptosis (Jeremias et al., 2000; Hotchkiss et al., 2009; Zhang et al., 2019a). MIRI increases mitochondrial permeability and caspase-9 expression level to activate caspase-3, caspase-6, and caspase-7 (Gottlieb and Engler, 1999; Hotchkiss et al., 2009). The mitochondria pathway is regulated by Bax and Bcl-2, which dissociates cytochrome C (cyt-C) and further activates caspase proteins (Hamacher-Brady et al., 2006; Hotchkiss et al., 2009). NF-κB can be transported to the nucleus by binding to IκB, thus causing apoptosis (Hayden and Ghosh, 2004). And NF-κB has function of inhibiting anti-apoptotic protein Bcl-2 (Neamatallah et al., 2018). MIRI up-regulates the levels of reactive oxygen species (ROS) and ER stress and further leads to intracellular calcium overload and apoptosis (Chaudhari et al., 2014; Lai et al., 2020; Song et al., 2020; Shi et al., 2021). ROS activates ER stress and JNK to increase the content of ROS (Chaudhari et al., 2014; Son et al., 2014; Lu et al., 2017; Chu et al., 2019). Increased ROS activates the PI3K/Akt signaling pathway, and the PI3K/Akt signaling pathway can regulate Nrf2 and eNOS to affect apoptosis (Gao et al., 2002; Syamsunarno et al., 2021; Zheng et al., 2021). MAPK contains JNK, p38, and ERK (Chang and Karin, 2001). JNK, p38, and ERK affect cell apoptosis via regulating c-Jun, MAPKAP-2, and Nrf2 (Davis, 2000; Ai et al., 2015; Zhang et al., 2019a). Figure 1 shows the apoptosis pathways in MIRI.

## Effects and Mechanisms of Ginsenosides on Myocardial Cell Apoptosis in Myocardial Ischemia/Reperfusion Injury

**FIGURE 2:**
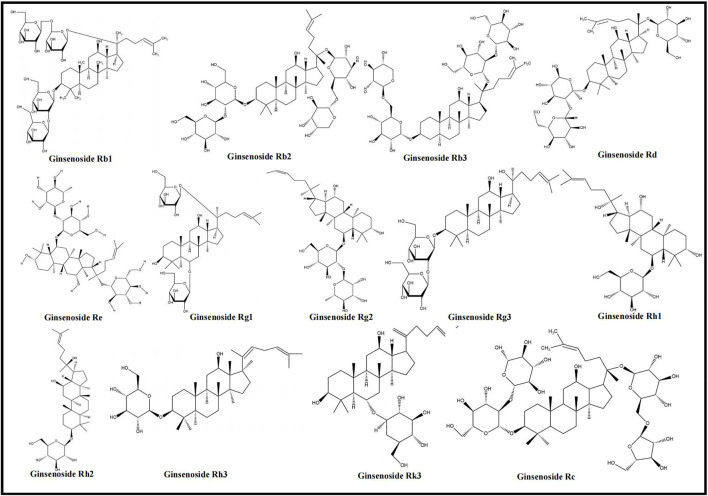
Chemical structures of ginsenosides (ginsenosides Rb1, Rb2, Rb3, Rd, Re, Rg1, Rg2, Rg3, Rh1, Rh2, Rh3, Rk3, and Rc).

*Panax ginseng*, a medicinal plant, belongs to the Araliaceae family and has a long history of usage (Geng et al., 2010). The main active ingredients of *Panax ginseng* are ginsenosides (Wang H. et al., 2021), which inhibit oxidative stress, enhance immune regulation, promote physiological functions (Chen S. et al., 2019; Wang H. et al., 2021), and are adopted to improve IHD, depression, diabetes, etc. (Zhang JH. et al., 2021; Wang Q. et al., 2021; Jiang et al., 2021). Ginsenosides Rb1, Rb2, Rb3, Rd, Re, Rg1, Rg2, Rg3, Rh1, Rh2, Rh3, Rk3, and Rc were proved to alleviate MIRI. The chemical structures of ginsenosides determine their pharmacological effects, especially hydroxyl groups and sugar moieties (Kim et al., 2015). Based on the differences in the parent ring structure, ginsenosides are divided into PPD (Ra1/2/3, Rb1/2/3, Rc, Rd, Rg3, Rh2, F2, compound K), oleanolic acid (Rh3, Ro, Ri), and PPT (Re, Rf, Rg1/2, Rh1, F1) types (Sun et al., 2016). The parent ring structure of PPD type ginsenosides contains two hydroxyl groups at C-3 and C-12, and their sugar moieties attach to β-OH at C-3 and/or C-20 (Bai et al., 2014; Kim et al., 2015). The oleanolic acid type ginsenosides are comprised of a pentacyclic structure with the aglycone oleanolic acid (Choi, 2008; Kim et al., 2015). The parent ring structure of PPT type ginsenosides contains three hydroxyl groups at C-3, C-6, and C-12, and sugar moieties attach to β-OH at C-20 and/or α-OH at C-6 (Bai et al., 2014; Kim et al., 2015). It was demonstrated that the three types of ginsenosides were effective in inhibiting MC apoptosis and regulating different apoptotic pathways to relieve MIRI. According to the included studies, PPD type ginsenosides can trigger the death receptor–mediated signaling pathway; PPD and PPT type ginsenosides both regulate PI3K/Akt–mediated and NF-κB–mediated signaling pathways; furthermore, all three types of ginsenosides can affect mitochondria- and MAPK-mediated signaling pathways. The chemical structures of the ginsenosides included in this study are shown in Figure 2. The mechanisms of ginsenosides on MC apoptosis in MIRI are summarized in Supplementary Table S1.

### Death Receptor–Mediated Signaling Pathway

**FIGURE 3:**
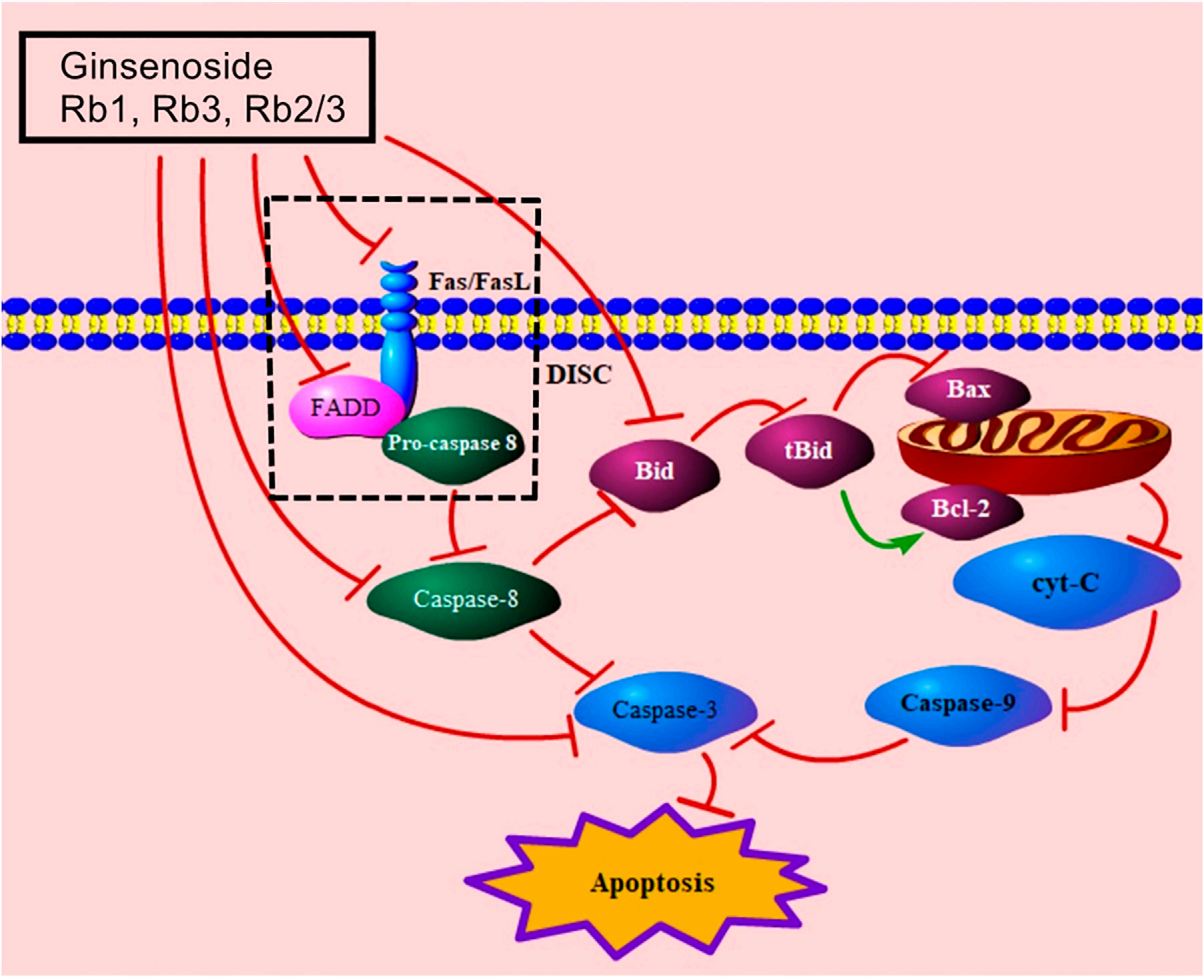
Death receptor–mediated signaling pathway of ginsenoside in relieving MC apoptosis. FasL, Fas ligand; FADD, Fas-associated death domain protein; DISC, death-induced signal complex; tBid, truncated Bid; cyt-C, cytochrome C.

The death receptor–mediated signaling pathway is the extrinsic pathway of apoptosis, induced by the binding of death receptors and their death ligands (Fas/FasL, TRAIL/TRAILR1, TRAIL/TRAILR2, TNF/TNFR1) (Galluzzi et al., 2012; Lee et al., 2012). Published studies addressing the death receptor–mediated MC apoptotic pathway of ginsenosides focus on the Fas/FasL signaling pathway. Fas/FasL binds to the FADD and transmits the apoptotic signal to procaspase-8, resulting in the formation of death-induced signal complex (DISC), which leads to caspase hydrolysis (Lee et al., 2012; Wang and Su, 2018). The combination of ginsenosides Rb3 and Rb2/Rb3 effectively regulates FasL and FADD to decrease the levels of caspase-8 and caspase-3 (Liu, 2014), while ginsenoside Rb1 improves MC apoptosis via the down-regulation of caspase-8 and caspase-3 (Ai et al., 2015). Moreover, caspase-8 can trigger the mitochondria-mediated signaling pathway by cleaving Bid (Chae et al., 2007). Bid decreases the level of Bcl-2 and increases the level of Bax to increase the release of cyt-C, and the levels of caspase-9 and caspase-3 (Chen et al., 2001; Pasdois et al., 2011; Kim et al., 2021). Ai et al. reported that ginsenoside Rb1 down-regulated caspase-8, bid, caspase-9, caspase-3, and cyt-C (Ai et al., 2015). The death receptor–mediated signaling pathway of ginsenosides in relieving MC apoptosis is shown in Figure 3.

### Mitochondria-Mediated Signaling Pathway

**FIGURE 4:**
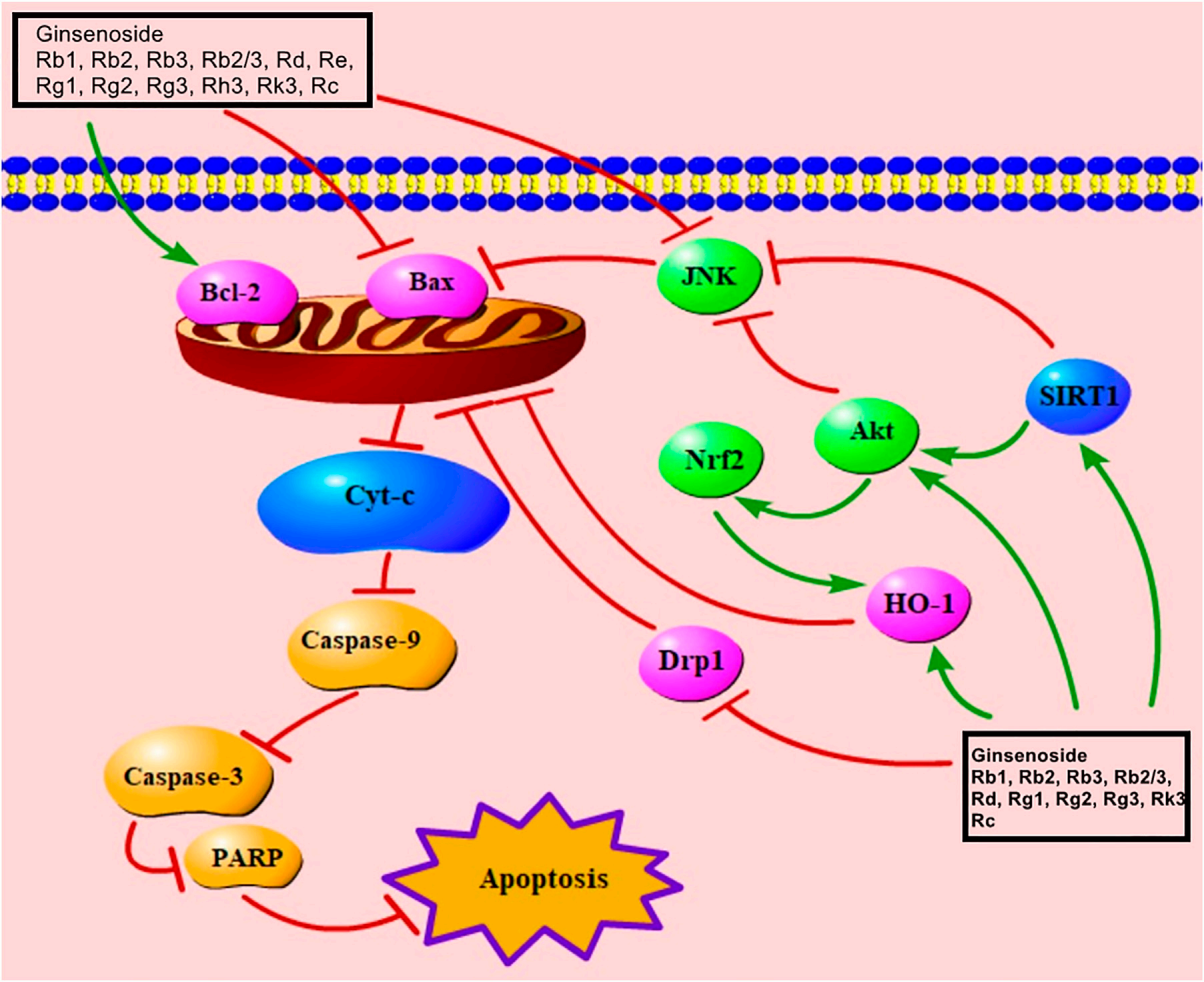
Mitochondria-mediated signaling pathway of ginsenoside in relieving MC apoptosis. Cyt-C, cytochrome C; PARP, poly(ADP-ribose) polymerase; Nrf2, nuclear factor E2–related factor 2; SIRT1, sirtuin 1; Drp1, dynamin-related protein 1; HO-1, heme oxygenase-1.

Mitochondria play a significant role in adjusting metabolism, generating ROS, and guaranteeing cell activity (Zhang et al., 2019a; Zhang X. et al., 2021). Bcl-2 inhibits apoptosis, whereas Bax promotes it, causing damage to the membrane structure and potential of mitochondria (Wang Q. et al., 2016). When the balance between Bcl-2 and Bax is disrupted, the mitochondrial membrane potential is reduced and the permeability of mitochondrial membrane is increased (Wang G. et al., 2021). Damaged mitochondria release cyt-C, which then increases the levels of caspase-9 and caspase-3 (Zhang et al., 2019a; Pu et al., 2013); increased caspase-3 up-regulates ADP-ribose polymerase (PARP), leading to apoptosis (Aggeli et al., 2021; Toit et al., 2020). Previous studies indicated that ginsenoside Rb1, Rb2, Rb3, Rb2/Rb3 combination, Rd, Re, Rg1, Rg2, Rg3, Rh3, Rk3, Rc all relieved MC apoptosis via regulating Bax, Bcl-2, cyt-C, caspase-9, caspase-3, and PARP (Supplementary Table S1). Decreased SIRT1 can reduce Akt to trigger the mitochondria-mediated signaling pathway by regulating JNK, Nrf2, and Bax (Ai et al., 2015; Pai et al., 2021). Nrf2, an important signaling molecule involved in cardioprotection (Zhu et al., 2008), regulates HO-1, which also has a cardioprotective effect (Liu et al., 2007; Zhu et al., 2008). The mitochondria-mediated signaling pathway, induced by ginsenosides Rb1, Rk3, and Rg3, is associated with the regulation of Akt, Nrf2, and HO-1 (Sun, 2013; Ai et al., 2015; Li L. et al., 2020). Ginsenosides Rb2 and Rg2 also up-regulate SIRT1 to trigger the mitochondria-mediated apoptotic pathway (Fu et al., 2018; Xue et al., 2020). Ginsenoside Rb3 was found to be effective in regulating Nrf2 (Sun et al., 2019). Additionally, previous studies showed that Rb2/Rb3 combination, Rd and Rg1 reduced mitochondria damage via increment of Akt (Wang et al., 2013; Liu, 2014; Qin et al., 2018). Dephosphorylated Drp1 is recruited to the mitochondrial outer membrane to cause damage to mitochondria (Yang, 2013). Ginsenoside Rb1 can inhibit the mRNA level of Drp1 (Yang, 2013). The mitochondria-mediated signaling pathway of ginsenosides in improving MIRI is presented in Figure 4.

### PI3K/Akt-Mediated Signaling Pathway

**FIGURE 5:**
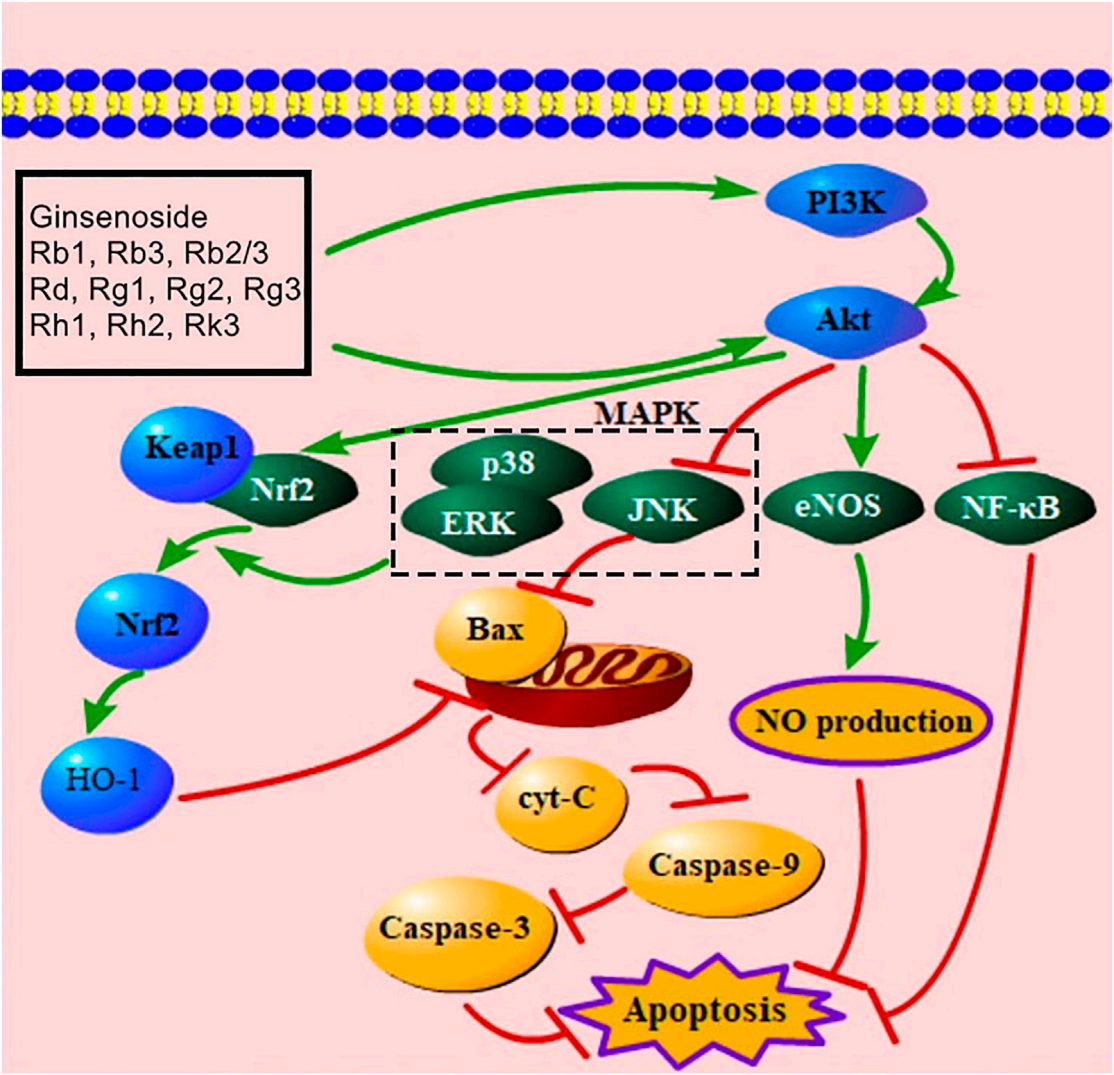
PI3K/Akt-mediated signaling pathway of ginsenoside in relieving MC apoptosis. PI3K, phosphatidylinositol-3-kinase; MAPK, mitogen-activated protein kinase; Keap1, Kelch-like ECH-associated protein 1; Nrf2, nuclear factor E2–related factor 2; HO-1, heme oxygenase-1; ERK, extracellular signal–regulated kinase; Cyt-C, cytochrome C; eNOS, endothelial nitric oxide synthase; NF-κB, nuclear factor of kappaB; NO, nitric oxide.

The PI3K/Akt signaling pathway is an important pathway by which ginsenosides improve apoptosis. The activation of this pathway promotes angiogenesis, alleviates tissue hypoxia, suppresses cell damage, and improves MC apoptosis (Wang M. et al., 2021; Zhang J. et al., 2021; Cao et al., 2021). Evidence showed that ginsenoside Rb1, Rb3, Rb2/Rb3 combination, Rd, Rg1, Rg2, Rg3, Rh1, Rh2, Rk3 activated the PI3K/Akt-mediated signaling pathway (Supplementary Table S1). Activated PI3K can phosphorylate Akt to regulate Nrf2, JNK, eNOS, and NF-κB for decreasing the number of apoptotic cells (Liu SX. et al., 2012; Ai et al., 2015; Liu et al., 2017; Luan et al., 2019; Fu Y. et al., 2021). When MIRI occurs, Nrf2 is released from Keap1 to the nucleus and activates HO-1 to alleviate apoptosis (Mann et al., 2007). Moreover, Nrf2 nuclear export is regulated by the MAPK-mediated signaling pathway (Mann et al., 2007). Ginsenosides Rb1 and Rk3 both increase Nrf2 by down-regulating JNK, ERK, and p38 MAPK (Sun, 2013; Ai et al., 2015). As mentioned above, the PI3K/Akt signaling pathway can decrease JNK to trigger the mitochondria-mediated signaling pathway for inhibiting apoptosis. A previous study presented that ginsenosides Rg2, Rg3, Rh1, and Rh2 up-regulated Akt and down-regulated JNK to inhibit apoptosis (Feng et al., 2017). Phosphorylated eNOS increases NO production and improves MC apoptosis (He et al., 2016), and ginsenosides Rb1, Rg1, and Rg3 can induce the phosphorylation of eNOS (Wang, 2008; Wang et al., 2015; Qin et al., 2018). In addition, ginsenosides Rb3 and Rg1 both up-regulate Akt to decrease NF-κB (Li, 2014; Ma et al., 2014). The PI3K/AKT/NF-κB ginsenoside pathway is considered an important mediator of cell survival and immune responses (Peng et al., 2013). This PI3K/Akt-mediated signaling pathway is presented in Figure 5.

### NF-κB–Mediated Signaling Pathway

**FIGURE 6:**
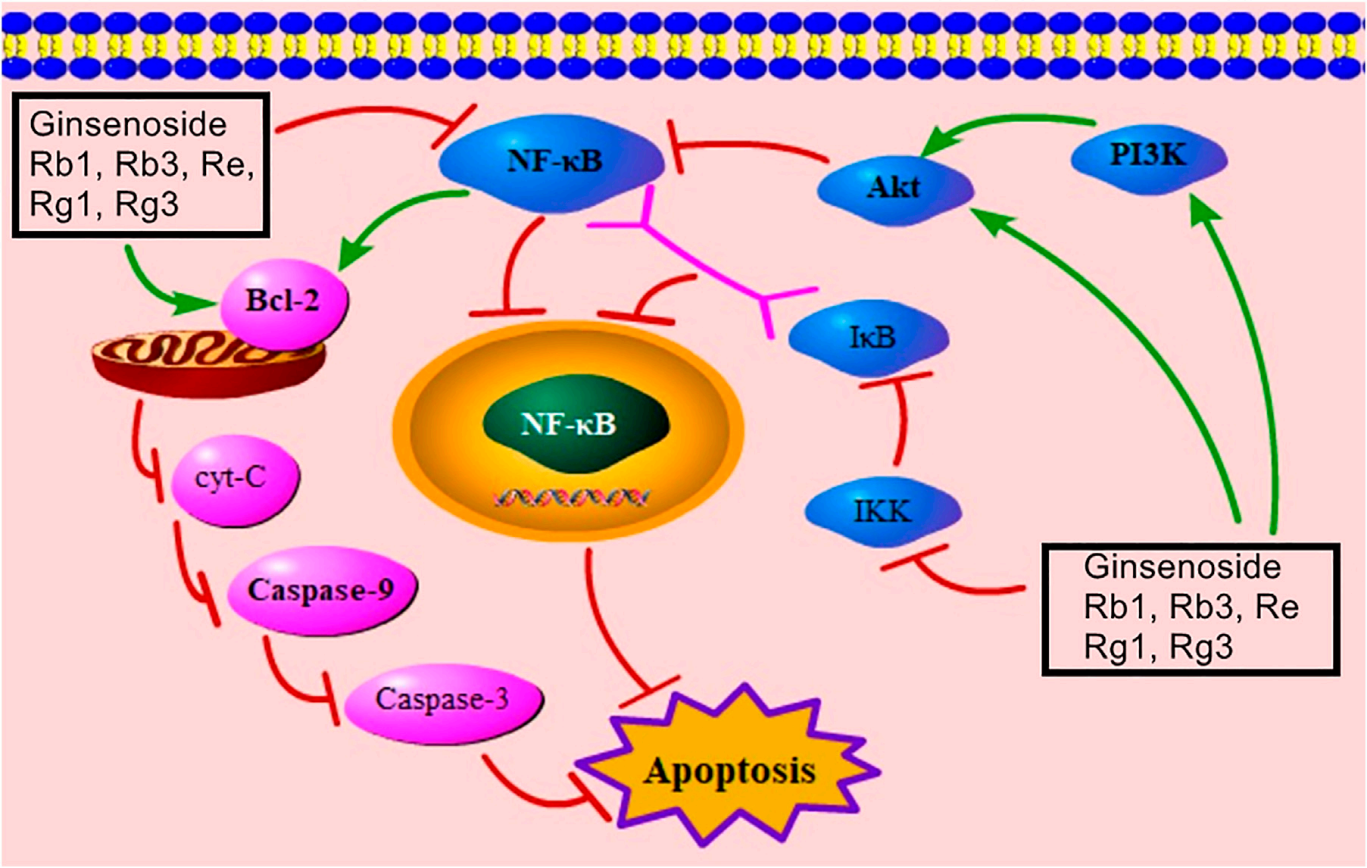
NF-κB–mediated signaling pathway of ginsenoside in relieving MC apoptosis. NF-κB, nuclear factor of kappaB; cyt-C, cytochrome C; PI3K, phosphatidylinositol-3-kinase; IκB, inhibitor of NF-κB; IKK, IκB kinase.

NF-κB belongs to a family of related transcription factors and participates in the regulation of immune responses, proinflammatory cytokines’ control, and cell death (Hussen et al., 2021; Hall et al., 2006). The Rel homology domain of NF-κB binds to IκB, and the complex of NF-κB/IκB inhibits the transport of NF-κB to the nucleus, thus inducing apoptosis (Hayden and Ghosh, 2004). Meanwhile, IκB is phosphorylated by IKK (Hayden and Ghosh, 2008). Recent studies have stated that ginsenosides Rb1, Rb3, Re, Rg1, and Rg3 can down-regulate IKKα, IκBα, and NF-κB, thus relieving MC apoptosis via inhibiting the NF-κB–mediated signaling pathway (Supplementary Table S1). Moreover, NF-κB can down-regulate Bcl-2 to trigger the mitochondria-mediated signaling pathway (Neamatallah et al., 2018; Duan et al., 2021). Figure 6 presents the NF-κB–mediated signaling pathway of ginsenoside in relieving MC apoptosis.

### MAPK-Mediated Signaling Pathway

**FIGURE 7:**
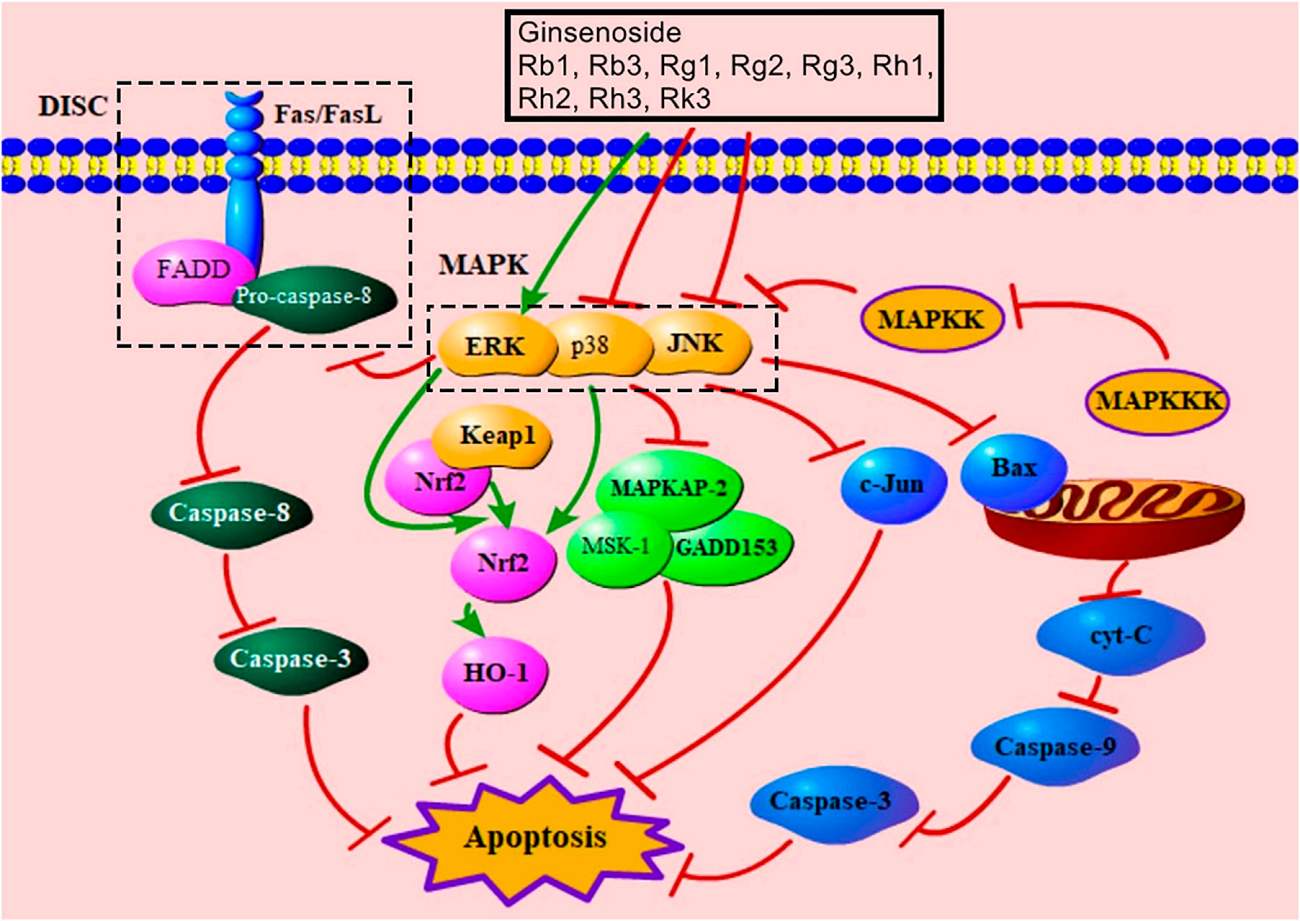
MAPK-mediated signaling pathway of ginsenoside in relieving MC apoptosis. DISC, death-induced signal complex; FasL, Fas ligand; FADD, Fas-associated death domain protein; MAPK, mitogen-activated protein kinase; ERK, extracellular signal–regulated kinase; Keap1, Kelch-like ECH-associated protein 1; Nrf2, nuclear factor E2–related factor 2; HO-1, heme oxygenase-1; MAPKAP-2, MAPK-activated protein kinase-2; MSK-1, mitogen- and stress-activated protein kinase; MAPKK, MAPK kinase; MAPKKK, MAPK kinase kinase; c-Jun, c-Jun N-terminal kinase; cyt-C, cytochrome C.

MAPK is an important signal transducing enzyme that has effects on the regulation of gene expression, cell proliferation, and cell death (Chang and Karin, 2001). MAPK kinase (MAPKK) is activated by MAPK kinase kinase (MAPKKK) to reactivate MAPK (Junttila et al., 2008). MAPK includes JNK, p38, and ERK, which are activated by special MAPKK and have different functions (Chang and Karin, 2001). The activation of JNK and p38 mediates apoptosis (Junttila et al., 2008). JNK promotes apoptosis through regulating c-Jun, which is its most classical substrate (Davis, 2000). JNK also effectively regulates pro-apoptotic protein, Bax (Syeda et al., 2019). The p38 MAPK pathway is related to the regulation of inflammation, gene expression, and energetic metabolism (Bassi et al., 2008). p38 participates in the promotion of apoptosis via its substrates, such as MAPKAP-2, MSK-1, and GADD153 (Zhang et al., 2019a; Ashraf et al., 2014; Das et al., 2006). Inhibition of the p38 MAPK pathway up-regulates the levels of Nrf2 and HO-1 to increase antioxidative proteins and improve apoptosis (Chen et al., 2021). Evidence indicated that ginsenosides Rb1, Rb3, Rg2, Rg3, Rh1, Rh2, and Rk3 had a function of down-regulating JNK, and ginsenosides Rb1 and Rk3 decreased the level of p38 (Supplementary Table S1). ERK can proliferate cells and regulate cell growth, and activated ERK inhibits the formation of DISC to relieve death receptor–mediated signaling pathway–induced apoptosis (Meloche and Pouysségur, 2007; Holmström et al., 2000). Additionally, ERK can increase Nrf2 to alleviate mitochondria damage (Ai et al., 2015). Ginsenosides Rb1, Rg1, and Rk3 can up-regulate ERK (Supplementary Table S1). Notably, one study indicated that ginsenoside Rb1 down-regulated ERK to inhibit apoptosis (Ai et al., 2015). In this study, MIRI increases the level of ERK. And over-expressed ERK leads to reversible or permanent cell cycle arrest (Meloche and Pouysségur, 2007); thus, ginsenoside Rb1 may decrease over-expressed ERK induced by apoptosis to protect MCs. Figure 7 showed MAPK-mediated signaling pathway of ginsenoside in relieving MC apoptosis.

### Other Pathways

In addition to the above-mentioned apoptotic signaling pathway, other pathways have been reported in previous studies. Li et al. reported that ginsenoside Rb1 improved MIRI by preserving PDH activity and inhibiting SDH activity (Li et al., 2017). Ginsenoside Rb1 also inhibits apoptosis by regulating microRNAs (miRNAs), namely, mir-208, mir-1, mir-29a, mir-21, and mir-320 (Yan et al., 2015; Yan et al., 2016). In 2012, Zhang et al. indicated that ginsenoside Rg1 increased ATP content and mTOR and decreased AMPKα, LC3B-1, and Beclin-1 to inhibit apoptosis and autophagy (Zhang et al., 2012). Moreover, ginsenoside Rg2 improves antioxidant enzyme activity (SOD, LDH, GXH-Px), and ginsenoside Rh3 increases SERCA (Zhou, 2009; Wang J. et al., 2016).

## Conclusion and Perspective

MIRI is functional and organic damage to the heart, which results from restoration of blood flow in ischemic areas (Wang K. et al., 2021). Through a number of studies addressing MIRI, the mechanisms of MIRI have not been fully revealed. Previous studies indicated that MC apoptosis was one of the fundamental pathogenic factors of MIRI, and the inhibition of MC apoptosis was effective in alleviating MIRI (Zhou et al., 2018; Xu et al., 2019; Fu D. et al., 2021). Ginsenosides can improve MIRI by relieving mitochondria damage, resisting oxidation, reducing inflammatory response, and inhibiting the generation of DISC (Wang and Roh, 2020; Shaukat et al., 2021). Ginsenosides can relieve MIRI via multiple signaling pathways, such as the death receptor signaling pathway, mitochondria signaling pathway, PI3K/Akt signaling pathway, NF-κB signaling pathway, and MAPK signaling pathway. The occurrence and development of MIRI is complex and multi-factor interacted; thus, it is vital to investigate multi-target therapy in future studies. Ginsenosides, which are regarded as undoubtedly low-toxicity drugs (Xu JF. et al., 2021), have favorable safety profiles (Mancuso and Santangelo, 2017). Toxicity studies showed that most ginsenosides have no oral toxicity, such as Re, Rg2, and Rh2 (Wang et al., 2006; Lu et al., 2012; Gou et al., 2020). Undeniably, in vitro studies indicated that ginsenosides Rb1, Rg1, and Re had embryotoxic and teratogenic effects (Chan et al., 2004; Liu et al., 2005; Liu et al., 2006). However, results from in vitro animal studies may not reflect the true conditions in humans; thus, previous studies suggested that these ginsenosides need to be used with caution in clinics during the first trimester of gestation, before more data in humans are available (Liu et al., 2006; Mancuso and Santangelo, 2017). Overall, the development and application of ginsenosides in improving MIRI are significant, and the toxicity data from *in vivo* studies and clinical studies are needed.

Currently, multiple studies have explored the anti-apoptotic mechanism of ginsenosides; however, problems still exist, and further studies are needed. Firstly, the research studies about the structure–function relationship of ginsenosides in inhibiting MIRI are still needed to be conducted. The hydroxyl groups and sugar moieties have influences on the pharmacological effects of ginsenosides, which can interact with membrane lipids (Kim et al., 2015). Thus, the research of structure–function relationship of ginsenosides in anti-MIRI can contribute to developing safe and effective drugs via chemical modification. Secondly, the current results are mainly generated by *ex vivo* experiments or animal experiments. Rare clinical evidence has showed that ginsenoside Rb has protective effects on MIRI in patients undergoing mitral valve surgery (Zhan et al., 1994). Existing studies are in infancy, and more clinical research studies are needed to be designed and conducted to supply further clinical evidence. Thirdly, evidence showed that pyroptosis occurred during the development of MIRI (Xu XN. et al., 2021; Ji et al., 2021), and pro-apoptotic caspase-3 can cleave GSDME to induce pyroptosis (Wang Y. et al., 2017). However, no study has confirmed the anti-pyroptosis effect of ginsenosides in improving MIRI. Thus, the mechanisms of ginsenosides need to be further explored.

Overall, this review of anti-apoptotic mechanisms of ginsenoside in MIRI presents pharmacological mechanisms and lays the foundation for further research studies, hoping to contribute to the development of undiscovered mechanism and new drugs.
