# Supplementary material for: Inhibition of Myocardial Cell Apoptosis Is Important Mechanism for Ginsenoside in the Limitation of Myocardial Ischemia/Reperfusion Injury
Source: Front Pharmacol. 2022 Mar 1;13:806216. doi: 10.3389/fphar.2022.806216 (PMC8921549; doi:10.3389/fphar.2022.806216)
Supplement: Supplementary file 1 [file Table1.pdf]

Table 1. The effects and mechanisms of ginsenoside in relieving MC apoptosis.

Potential pathway	Compound	Detail mechanism	Concentration / Dosage	Duration	Animal / Cell	References
Death receptors mediated signaling pathway	Ginsenoside Rb1	Down-regulating caspase-3, caspase-8, Bid, cyt-C, caspase-9.	3.125 μg/ml; 6.25 μg/ml; 12.5 μg/ml	1 time	H9c2 cell, ex vivo	(Ai *et al.*, 2015)
	Ginsenoside Rb3	Down-regulating caspase-3, caspase-8, FasL, FADD.	100 μg/ml; 200 μg/ml.	1 time	H9c3 cell, ex vivo	(Liu, 2014)
	Ginsenoside Rb2/Rb3	Down-regulating caspase-3, caspase-8, FasL, FADD.	100 μg/ml; 200 μg/ml.	1 time	H9c4 cell, ex vivo	(Liu, 2014)
Mitochondria mediated signaling pathway	Ginsenoside Rb1	Up-regulating MMP; down-regulating cyt-C, cleaved caspase-3, the opening of mPTP.	100 μM	1 time	H9c2 cell, ex vivo	(Zhang, 2020)
	Ginsenoside Rb1	Down-regulating the level of mitochondria division, Drp1.	50 μM; 200 μM	1 time	SD rats, ex vivo	(Yang, 2013)
	Ginsenoside Rb1	Up-regulating Bcl-2, PARP1/2, p-Akt, Nrf2, HO-1; down-regulating Bax, cyt-C, caspase-9, caspase-3, p-JNK.	3.125 μg/ml; 6.25 μg/ml; 12.5 μg/ml	1 time	H9c2 cell, ex vivo	(Ai *et al.*, 2015)
	Ginsenoside Rb1	Down-regulating cyt-C, cleaved caspase-3, mPTP.	50 μM; 100 μM; 150 μM; 200 μM	1 time	H9c2 cell, ex vivo	(Zhang *et al.*, 2019)
	Ginsenoside Rb1	Up-regulating Bcl-2, p-Akt, down-regulating cleaved caspase-3.	40 mg/kg, i.v.	1 time	SD rats, in vivo	(Wu, 2011)
	Ginsenoside Rb1	Up-regulating Bcl-2/Bax, p-Akt, down-regulating caspase-3.	6 mg/kg, i.p.	1 time	ICR mice, in vivo	(Yang *et al.*, 2021)

Ginsenoside Rb1	Up-regulating Bcl-2, Bcl-2/Bax; down-regulating cleaved caspase-3, Bax.	20 mg/kg, i.p.; 40 mg/kg, i.p.; 80 mg/kg, i.p.	3 days (1 time/day)	SD rats, in vivo	(Li *et al.*, 2020a)
Ginsenoside Rb1	Down-regulating Bax/Bcl-2, cleaved caspase-9, cleaved caspase-3.	5 mg/kg/h, i.v., 30 min.	1 time	SD rats, in vivo	(Cui *et al.*, 2017)
Ginsenoside Rb2	Up-regulating Bcl-2, procaspase-3, procaspase-9, SIRT1; down-regulating Bax, cleaved caspase-9, cleaved caspase-3.	10 mg/kg, p.o.; 20 mg/kg, p.o.	3 days (1 time/day)	Wistar rats, in vivo	(Xue *et al.*, 2020)
Ginsenoside Rb2	Up-regulating Bcl-2, procaspase-9, procaspase-3.	10 mg/kg, p.o.; 20 mg/kg, p.o.	3 days (1 time/day)	SD rats, in vivo	(Fu *et al.*, 2016)
Ginsenoside Rb3	Up-regulating Bcl-2; down-regulating Bax, cyt-C, caspase-3, caspase-9.	100 μg/ml; 200 μg/ml.	1 time	H9c3 cell, ex vivo	(Liu, 2014)
Ginsenoside Rb3	Up-regulating Bcl-2, PPAR; down-regulating Bax.	40 μM	1 time	H9c2 cell, ex vivo	(Chen *et al.*, 2019)
Ginsenoside Rb3	Up-regulating Bcl-2; down-regulating Bax.	20 mg/kg, p.o.	3 days (1 time/day)	SD rats, in vivo	(Liu *et al.*, 2014)
Ginsenoside Rb3	Up-regulating Nrf2; down-regulating Bax/Bcl-2.	50 mg/kg, p.o.	5 days (1 time/day)	SD rats, in vivo	(Sun *et al.*, 2019)
Ginsenoside Rb3	Up-regulating Bcl-2; down-regulating Bax, Bax/Bcl-2, caspase-3.	20 mg/kg, i.v.	3 days (1 time/day)	SD rats, in vivo	(Liu *et al.*, 2020)
Ginsenoside Rb2/Rb3	Up-regulating Bcl-2, p-AKT; down-regulating Bax, cyt-C, caspase-3, caspase-9.	100 μg/ml; 200 μg/ml.	1 time	H9c3 cell, ex vivo	(Liu, 2014)

Ginsenoside Rb2/Rb3	Up-regulating Bcl-2; down-regulating Bax, Bax/Bcl-2, caspase-3.	20 mg/kg, i.v.	3 days (1 time/day)	SD rats, in vivo	(Liu *et al.*, 2020)
Ginsenoside Rd	Up-regulating Bcl-2/Bax, MMP, p-AKT; down-regulatin cyt-C, caspase-9, caspase-3.	50 mg/kg, i.v.	1 time	SD rats, in vivo	(Wang *et al.*, 2013)
Ginsenoside Re	Down-regulating Bax.	20 mg/kg, i.v.	1 time	Wistar rats, in vivo	(Liu *et al.*, 2002)
Ginsenoside Rg1	Up-regulating Bcl-2; down-regulating Bax, Cytc.	30 mg/kg, p.o.	7 days (1 time/day)	SD rats, in vivo	(Zhao *et al.*, 2021)
Ginsenoside Rg1	Up-regulating Bcl-2; down-regulating Bax, cleaved caspase-9, cleaved caspase-3, cleaved PARP.	20 mg/kg, i.p.; 40 mg/kg, i.p.	14 days (1 time/day)	Wistar rats, in vivo	(Shen *et al.*, 2017)
Ginsenoside Rg1	Up-regulating Bcl-2, Akt; down-regulating Bax, caspase-3, caspase-9.	50 μM; 100 μM; 150 μM; 200 μM.	1 time	H9c2 cell, ex vivo	(Qin *et al.*, 2018)
Ginsenoside Rg1	Up-regulating MMP-2; down-regulating Bax/Bcl-2, caspase-3, caspase-9.	35 mg/kg, i.v.	1 time	Rat, in vivo	(Yuan *et al.*, 2019)
Ginsenoside Rg1	Down-regulating Bax/Bcl-2, caspase-3.	5 mg/kg/h, i.v., 30 min.	1 time	SD rats, in vivo	(Li *et al.*, 2018)
Ginsenoside Rg1	Down-regulating Bax/Bcl-2, cleaved caspase-3.	1 mg/kg, i.v.; 5 mg/kg, i.v.; 10 mg/kg, i.v.	1 time	SD rats, in vivo	(Li, 2014)
Ginsenoside Rg1	Up-regulating Bcl-2; down-regulating Bax.	100 mg/kg, p.o.	10 days (1 time/day)	SD rats, in vivo	(Qian *et al.*, 2019)
Ginsenoside Rg2	Up-regulating Bcl-2, procaspase-9, procaspase-3, SIRT1; down-regulating Bax.	10 mg/kg, p.o.; 20 mg/kg, p.o.	7 days (1 time/day)	SD rats, in vivo	(Fu *et al.*, 2018)
Ginsenoside Rg3	Up-regulating Bcl-2; down-regulating Bax, caspase-3.	5 mg/kg, p.o.; 20 mg/kg, p.o.	7 days (1 time/day)	SD rats, in vivo	(Zhang *et al.*, 2016)

	Ginsenoside Rg3	Up-regulating Bcl-2, Bcl-2/Bax, p-Akt; down-regulating Bax, caspase-9, caspase-3.	10 mM	1 time	SD rats, ex vivo	(Wang *et al.*, 2015)
	Ginsenoside Rg3	Up-regulating Bcl-2; down-regulating Bax, cleaved caspase-3.	5 mg/kg, p.o.; 20 mg/kg, p.o.	7 days (1 time/day)	SD rats, in vivo	(Zhang, 2017)
	Ginsenoside Rg3	Up-regulating Bcl-2, SIRT1, Nrf2, HO-1; down-regulating Bax, caspase-3.	0.5 mg, i.m.	1 time	H9c2 cell, ex vivo	(Li *et al.*, 2020b)
	Ginsenoside Rh3	Up-regulating Bcl-2; down-regulating Bax.	4 mg/kg; 8 mg/kg; 16 mg/kg	7 days (1 time/day)	SD rats, in vivo	(Wang *et al.*, 2017a)
	Ginsenoside Rh3	Up-regulating Bcl-2; down-regulating Bax.	40 mg/kg	7 days (1 time/day)	SD rats, in vivo	(Wang *et al.*, 2019)
	Ginsenoside Rk3	Up-regulating Bcl-2, Akt, Nrf2, HO-1; down-regulating Bax, caspase-3.	12.5 μg/ml	1 time	H9c2 cell, ex vivo	(Sun, 2013)
	Ginsenoside Rk3	Up-regulating Bcl-2/Bsx, p-AKT; down-regulating caspase-3.	6.25 μg/ml; 12.5 μg/ml; 25 μg/ml; 50 μg/ml.	1 time	H9c2 cell, ex vivo	(Sun *et al.*, 2013)
	Ginsenoside Rc	Up-regulating Bcl-2, SIRT1, Nrf2; down-regulating Bax.	10 mg/kg	14 days (1 time/day)	SD rats, in vivo	(Huang *et al.*, 2021)
PI3K/Akt mediated signaling pathway	Ginsenoside Rb1	Up-regulating p-Akt, p-eNOS.	40 mg/kg, i.v.	1 time	SD rats, in vivo	(Wu, 2011)
	Ginsenoside Rb1	Up-regulating p-Akt, p-eNOS.	40 mg/kg, i.v.	1 time	SD rats, in vivo	(Wang, 2008)
	Ginsenoside Rb1	Up-regulating p-Akt.	40 mg/kg	1 time	SD rats, in vivo	(Liu *et al.*, 2012)
	Ginsenoside Rb1	Up-regulating p-Akt.	40 mg/kg, i.v.	1 time	SD rats, in vivo	(Wu *et al.*, 2011)

Ginsenoside Rb1	Up-regulating p-Akt.	6 mg/kg, i.p.	1 time	ICR mice, in vivo	(Yang *et al.*, 2021)
Ginsenoside Rb1	Up-regulating p-Akt, nrf2; down-regulating p-JNK and ERK.	3.125 μg/ml; 6.25 μg/ml; 12.5 μg/ml	1 time	H9c2 cell, ex vivo	(Ai *et al.*, 2015)
Ginsenoside Rb3	Up-regulating p-Akt; down-regulating JNK, NF-κB.	2 μM; 5 μM.	1 time	H9c2 cell, ex vivo	(Ma *et al.*, 2014)
Ginsenoside Rb2/Rb3	Up-regulating p-Akt.	20 mg/kg, i.g.	3 days (1 time/day)	Wistar rats, in vivo	(Liu, 2014)
Ginsenoside Rd	Up-regulating p-Akt.	50 mg/kg, i.v.	1 time	SD rats, in vivo	(Wang *et al.*, 2013)
Ginsenoside Rg1	Up-regulating p-PI3K, p-Akt; down-regulating NF-κB.	1 mg/kg, i.v.; 5 mg/kg, i.v.; 10 mg/kg, i.v.	1 time	SD rats, in vivo	(Li, 2014)
Ginsenoside Rg1	Up-regulating p-PI3K, p-Akt, p-eNOS.	50 μM; 100 μM; 150 μM; 200 μM.	1 time	H9c2 cell, ex vivo	(Qin *et al.*, 2018)
Ginsenoside Rg2	Up-regulating p-Akt; down-regulating JNK.	2 μM	1 time	H9c2 cell, ex vivo	(Feng *et al.*, 2017)
Ginsenoside Rg3	Up-regulating p-Akt; down-regulating JNK.	2 μM	1 time	H9c2 cell, ex vivo	(Feng *et al.*, 2017)
Ginsenoside Rg3	Up-regulating p-Akt, p-eNOS.	10 mM	1 time	SD rats, ex vivo	(Wang *et al.*, 2015)
Ginsenoside Rh1	Up-regulating p-Akt; down-regulating JNK.	2 μM	1 time	H9c2 cell, ex vivo	(Feng *et al.*, 2017)
Ginsenoside Rh2	Up-regulating p-Akt; down-regulating JNK.	2 μM	1 time	H9c2 cell, ex vivo	(Feng *et al.*, 2017)

	Ginsenoside Rk3	Up-regulating p-Akt; down-regulating JNK.	6.25 μg/ml; 12.5 μg/ml; 25 μg/ml; 50 μg/ml.	1 time	H9c2 cell, ex vivo	(Sun *et al.*, 2013)
	Ginsenoside Rk3	Up-regulating p-Akt, Nrf2, HO-1; down-regulating JNK and p38MAPK.	12.5 μg/ml	1 time	H9c2 cell, ex vivo	(Sun, 2013)
NF-κB mediated signaling pathway	Ginsenoside Rb1	Down-regulating IKKα, IκBα.	6 mg/kg, i.p.	1 time	ICR mice, in vivo	(Yang *et al.*, 2021)
	Ginsenoside Rb3	Down-regulating NF-κB, IκBα.	2 μM; 5 μM.	1 time	H9c2 cell, ex vivo	(Ma *et al.*, 2014)
	Ginsenoside Re	Down-regulating NFκB-p65.	10 mg/kg, i.v.; 20 mg/kg, i.v.	1 time	Wistar rats, in vivo	(Cao, 2004)
	Ginsenoside Rg1	Down-regulating nucleus NFκB-p65.	1 mg/kg, i.v.; 5 mg/kg, i.v.; 10 mg/kg, i.v.	1 time	SD rats, in vivo	(Li, 2014)
	Ginsenoside Rg1	Down-regulating NFκB-p65.	5 mg/kg, p.o.; 10 mg/kg, p.o.	15 days (1 time/day)	SD rats, in vivo	(Fan *et al.*, 2015)
	Ginsenoside Rg3	Down-regulating NF-κB; IκBα,	0.5 mg, i.m.	1 time	H9c2 cell, ex vivo	(Li *et al.*, 2020b)
MAPK mediated signaling pathway	Ginsenoside Rb1	Down-regulating p38.	40 mg/kg, i.v.	1 time	SD rats, in vivo	(Wang and Zhang, 2016)
	Ginsenoside Rb1	Up-regulating ERK1/2.	6 mg/kg, i.p.	1 time	ICR mice, in vivo	(Yang *et al.*, 2021)
	Ginsenoside Rb1	Down-regulating p-JNK, ERK.	3.125 μg/ml; 6.25 μg/ml; 12.5 μg/ml	1 time	H9c2 cell, ex vivo	(Ai *et al.*, 2015)
	Ginsenoside Rb1	Down-regulating p38.	40 mg/kg, i.v.	1 time	SD rats, in vivo	(Li *et al.*, 2016)

	Ginsenoside Rb3	Down-regulating JNK.	2 μM; 5 μM.	1 time	H9c2 cell, ex vivo	(Ma *et al.*, 2014)
	Ginsenoside Rg1	Up-regulating ERK.	35 mg/kg, i.v.	1 time	Rat, in vivo	(Yuan *et al.*, 2019)
	Ginsenoside Rg2	Down-regulating JNK.	2 μM	1 time	H9c2 cell, ex vivo	(Feng *et al.*, 2017)
	Ginsenoside Rg3	Down-regulating JNK.	2 μM	1 time	H9c2 cell, ex vivo	(Feng *et al.*, 2017)
	Ginsenoside Rh1	Down-regulating JNK.	2 μM	1 time	H9c2 cell, ex vivo	(Feng *et al.*, 2017)
	Ginsenoside Rh2	Down-regulating JNK.	2 μM	1 time	H9c2 cell, ex vivo	(Feng *et al.*, 2017)
	Ginsenoside Rh3	Down-regulating p38.	0.5 mg/kg, p.o.	14 days (1 time/day)	SD rats, in vivo	(Fu *et al.*, 2020)
	Ginsenoside Rh3	Down-regulating p38.	0.5 mg/kg, p.o.	14 days (1 time/day)	SD rats, in vivo	(Cao *et al.*, 2020)
	Ginsenoside Rk3	Down-regulating JNK, p38; up-regulating ERK.	12.5 μg/ml	1 time	H9c2 cell, ex vivo	(Sun, 2013)
	Ginsenoside Rk3	Down-regulating JNK, p38; up-regulating ERK.	6.25 μg/ml; 12.5 μg/ml; 25 μg/ml; 50 μg/ml.	1 time	H9c2 cell, ex vivo	(Sun *et al.*, 2013)
Others	Ginsenoside Rb1	Improving PDH activity; inhibited SDH activity	50 mg/kg, p.o.	8 weeks (1 time/day)	ICR mice, in vivo	(Li *et al.*, 2017)
	Ginsenoside Rb1	Down-regulating mir-208.	40 μM	1 time	SD rats, ex vivo	(Yan *et al.*, 2016)

Ginsenoside Rb1	Down-regulating mir-1, mir-29a, mir-208; up-regulating mir-21, mir-320.	40 μM	1 time	SD rats, ex vivo	(Yan *et al.*, 2015)
Ginsenoside Rb1	Improving cell ultrastructure, reducing cell apoptosis.	0.01 μM; 0.1 μM; 1 μM; 10 μM	1 time	SD rats, ex vivo	(Bao *et al.*, 2010)
Ginsenoside Rb1	Reducing apoptotic cell.	20 mg/kg, i.v.	1 time	Wistar rats, in vivo	(Guan *et al.*, 2002)
Ginsenoside Rb1	Decreasing DNA cleavage, cell apoptosis.	0.4 mg, i.v.	1 time	Wistar rats, in vivo	(Zhang *et al.*, 2002)
Ginsenoside Re	Down-regulating caspase-9, caspase-3.	10 mg/kg, i.v.; 20 mg/kg, i.v.	2 times	Wistar rats, in vivo	(Lu, 2009)
Ginsenoside Re	Down-regulating caspase-3.	30 mg/kg, i.v.	1 time	Wistar rats, in vivo	(Gao *et al.*, 2011)
Ginsenoside Rg1	Up-regulating ATP content and mTOR; down-regulating AMPKα, LC3B-1 and Beclin-1.	100 μM	1 time	H9c2 cell, ex vivo	(Zhang *et al.*, 2012)
Ginsenoside Rg1	Improving cell ultrastructure, reducing cell apoptosis.	0.01 μM; 0.1 μM; 1 μM; 10 μM	1 time	SD rats, ex vivo	(Bao *et al.*, 2010)
Ginsenoside Rg1	Down-regulating cleaved caspase-3.	100 μM	1 time	H9c2 cell, ex vivo	(Xin, 2020)
Ginsenoside Rg2	Up-regulating SOD, GXH-Px; down-regulating LDH.	25 μM	1 time	Wistar rats, ex vivo	(Zhou, 2009)
Ginsenoside Rg2	Lowering apoptotic cell peak, reducing DNA breakage and cell apoptosis.	1 mg/kg, i.v.; 2 mg/kg, i.v.	1 time	Wistar rats, in vivo	(Tian *et al.*, 2004)
Ginsenoside Rh3	Up-regulating SERCA.	16 mg/kg, s.c.	7 days (1 time/day)	SD rats, in vivo	(Wang *et al.*, 2016)

Ginsenoside Rh3	Down-regulating caspase-3.	4 mg/kg	7 days (1 time/day)	SD rats, in vivo	(Wang *et al.*, 2017b)

References

[1] Ai, Q., Sun, G., Luo, Y., Dong, X., Hu, R., Meng, X., and Sun, X. (2015) Ginsenoside Rb1 prevents hypoxia-reoxygenationinduced apoptosis in H9c2 cardiomyocytes via an estrogen receptor-dependent crosstalk among the Akt, JNK, and ERK 1/2 pathways using a label-free quantitative proteomics analysis. RSC Adv. 5: 26346–26363. doi: 10.1039/c5ra02432c.

[2] Liu, X. (2014) The study on the protective effects and mechanisms of ginsenoside Rb3 and Rb2 combination on myocardial ischemia reperfusion injury. [Doctor's thesis]. [Changchun (China)]: Jilin University of China.

[3] Zhang, H. (2020) Ginsenoside Rb1 attenuates HR injury of H9c2 cells through mitochondrial ATP-sensitive potassium channels. [Master's Thesis]. [Jinzhou (China)]: Jinzhou Medical University.

[4] Yang, Y. (2013) Influences of ginsenoside Rbi on mitochondrial dynamics in NRVMs hypoxia/reoxyenation model. [Master's Thesis]. [Shantou (China)]: Shantou University.

[5] Zhang, H., Wang, X., Ma, Y., and Shi, Y. (2019) The Effect of Ginsenoside RB1, Diazoxide, and 5-Hydroxydecanoate on Hypoxia-Reoxygenation Injury of H9C2 Cardiomyocytes. Evid Based Complement Alternat Med. 2019: 6046405. doi: 10.1155/2019/6046405.

[6] Wu, Y. (2011) An experimental study on the effects of ginsenoside Rb1 against myocardial ischemia/reperfusion injury in streptozotocin-induced diabetic rats. [Doctor's Thesis]. [Wuhan (China)]: Wuhan University.

[7] Yang, W., Lai, Q., Zhang, L., Zhang, Y., Zhang, Y., Yu, B., Li, F., and Kou, J. (2021) Mechanisms dissection of the combination GRS derived from ShengMai preparations for the treatment of myocardial ischemia/reperfusion injury. J Ethnopharmacol. 264: 113381. doi: 10.1016/j.jep.2020.113381.

[8] Li, C.Y., Yang, P., Jiang, Y.L., Lin, Z., Pu, Y.W., Xie, L.Q., Sun, L., and Lu, D. (2020a) Ginsenoside Rb1 attenuates cardiomyocyte apoptosis induced by myocardial ischemia reperfusion injury through mTOR signal pathway. Biomed Pharmacother. 125: 109913. doi: 10.1016/j.biopha.2020.109913.

[9] Cui, Y.C., Pan, C.S., Yan, L., Li, L., Hu, B.H., Chang, X., Liu, Y.Y., Fan, J.Y., Sun, K., Li, Q., and Han, J.Y. (2017) Ginsenoside Rb1 protects against ischemia/reperfusion-induced myocardial injury via energy metabolism regulation mediated by RhoA signaling pathway. Sci Rep.

7: 44579. doi: 10.1038/srep44579.
[10] Xue, Y., Fu, W., Liu, Y., Yu, P., Sun, M., Li, X., Yu, X., and Sui, D. (2020) Ginsenoside Rb2 alleviates myocardial ischemia/reperfusion injury in rats through SIRT1 activation. J Food Sci. 85: 4039-4049. doi: 10.1111/1750-3841.15505.
[11] Fu, W., Yu, X., Lu, Z., Sun, F., Wang, Y., Zhang, Y., Zhang, Y., Chen, Y., Xu, H., and Sui, D. (2016) Protective effects of ginsenoside Rb2 on myocardial ischemia in vivo and in vitro. Int J Clin Exp Med. 9: 9843-9855.
[12] Chen, X., Wang, Q., Shao, M., Ma, L., Guo, D., Wu, Y., Gao, P., Wang, X., Li, W., Li, C., and Wang, Y. (2019) Ginsenoside Rb3 regulates energy metabolism and apoptosis in cardiomyocytes via activating PPARα pathway. Biomed Pharmacother. 120: 109487. doi: 10.1016/j.biopha.2019.109487.
[13] Liu, X., Jiang, Y., Yu, X., Fu, W., Zhang, H., and Sui, D. (2014) Ginsenoside-Rb3 protects the myocardium from ischemia-reperfusion injury via the inhibition of apoptosis in rats. Exp Ther Med. 8: 1751-1756. doi: 10.3892/etm.2014.2007.
[14] Sun, J., Yu, X., Huangpu, H., and Yao, F. (2019) Ginsenoside Rb3 protects cardiomyocytes against hypoxia/reoxygenation injury via activating the antioxidation signaling pathway of PERK/Nrf2/HMOX1. Biomed Pharmacother. 109: 254-261. doi: 10.1016/j.biopha.2018.09.002.
[15] Liu, X., Jiang, Y., Fu, W., Yu, X., and Sui, D. (2020) Combination of the ginsenosides Rb3 and Rb2 exerts protective effects against myocardial ischemia reperfusion injury in rats. Int J Mol Med. 45: 519-531. doi: 10.3892/ijmm.2019.4414.
[16] Wang, Y., Li, X., Wang, X., Lau, W., Wang, Y., Xing, Y., Zhang, X., Ma, X., and Gao, F. (2013) Ginsenoside Rd attenuates myocardial ischemia/reperfusion injury via Akt/GSK-3β signaling and inhibition of the mitochondria-dependent apoptotic pathway. PLoS One. 8: e70956. doi: 10.1371/journal.pone.0070956.
[17] Liu, Z., Li, Z., and Liu, X. (2002) Effect of ginsenoside Re on cardiomyocyte apoptosis and expression of Bcl-2/Bax gene after ischemia and reperfusion in rats. J Huazhong Univ Sci Technolog Med Sci. 22: 305-309. doi: 10.1007/BF02896771.
[18] Zhao, H., Zhang, D., Pang, L., Zhang, H., and Yu, R. (2021) Effects of astragaloside IV and ginsenoside Rg1 on myocardial apoptosis after myocardial ischemia reperfusion injury hyperlipidemia rats. Liaoning Journal of Traditional Chinese Medicine. 48: 188-191.
[19] Shen, W., Li, Y., and Yang, S. (2017) Effects of ginsenoside Rg1 on arrhythmias induced by ischemic/reperfusion injury in rats. Journal of Clinical Cardiology. 33: 465-469.
[20] Qin, L., Fan, S., Jia, R., and Liu, Y. (2018) Ginsenoside Rg1 protects cardiomyocytes from hypoxia-induced injury through the

PI3K/AKT/mTOR pathway. Pharmazie. 73: 349-355. doi: 10.1691/ph.2018.8329.
[21] Yuan, C., Wang, H., and Yuan, Z. (2019) Ginsenoside Rg1 inhibits myocardial ischaemia and reperfusion injury via HIF-1 α-ERK signalling pathways in a diabetic rat model. Pharmazie. 74: 157-162. doi: 10.1691/ph.2019.8858.
[22] Li, L., Pan, C.S., Yan, L., Cui, Y.C., Liu, Y.Y., Mu, H.N., He, K., Hu, B.H., Chang, X., Sun, K., Fan, J.Y., Huang, L., and Han, J.Y. (2018) Ginsenoside Rg1 Ameliorates Rat Myocardial Ischemia-Reperfusion Injury by Modulating Energy Metabolism Pathways. Front Physiol. 9: 78. doi: 10.3389/fphys.2018.00078.
[23] Li, L. (2014) The effect and mechanism of ginsenoside Rg1 in improving myocardial Ischemia/Reperfusion Injury. [Beijing (China)]: Beijing University of Chinese Medicine.
[24] Qian, S., Kan, J., and Yang, Y. (2019) Protective Effects of the Combination of Ginsenoside Rgl and Resveratrol Against Ischemia Reperfusion Injury of Cardiomyocytes. Journal of Liaoning University of TCM. 21: 47-49.
[25] Fu, W., Xu, H., Yu, X., Lyu, C., Tian, Y., Guo, M., Sun, J., and Sui, D. (2018) 20(S)-Ginsenoside Rg2 attenuates myocardial ischemia/reperfusion injury by reducing oxidative stress and inflammation: role of SIRT1. RSC Adv. 8: 23947-23962. doi: 10.1039/C8RA02316F.
[26] Zhang, L.P., Jiang, Y.C., Yu, X.F., Xu, H.L., Li, M., Zhao, X.Z., and Sui, D.Y. (2016) Ginsenoside Rg3 Improves Cardiac Function after Myocardial Ischemia/Reperfusion via Attenuating Apoptosis and Inflammation. Evid Based Complement Alternat Med. 2016: 6967853. doi: 10.1155/2016/6967853.
[27] Wang, Y., Hu, Z., Sun, B., Xu, J., Jiang, J., and Luo, M. (2015) Ginsenoside Rg3 attenuates myocardial ischemia/reperfusion injury via Akt/endothelial nitric oxide synthase signaling and the B-cell lymphoma/B-cell lymphoma-associated X protein pathway. Mol Med Rep. 11: 4518-4524. doi: 10.3892/mmr.2015.3336.
[28] Zhang, L. (2017) Effects and mechanism of alprostadil and ginsenoside Rg3 on myocardial ischemia/reperfusion injury in rats. [Doctor's Thesis]. [Jilin (China)]: Jinlin University.
[29] Li, L., Wang, Y., Guo, R., Li, S., Ni, J., Gao, S., Gao, X., Mao, J., Zhu, Y., Wu, P., Wang, H., Kong, D., Zhang, H., Zhu, M., and Fan, G. (2020b) Ginsenoside Rg3-loaded, reactive oxygen species-responsive polymeric nanoparticles for alleviating myocardial ischemia-reperfusion injury. J Control Release. 317: 259-272. doi: 10.1016/j.jconrel.2019.11.032.
[30] Wang, J., Cui, Y., Wang, J., Li, H., Dai, J., Ma, H., and Li, S. (2017a) Ginseng saponin Rh3 pretreatment to protect myocardial ischemia-

reperfusion injury in rats. Chinese Archives of Traditional Chinese Medicine. 35: 2783-2786.
[31] Wang, J., Cui, Y., Wang, J., Li, H., Dai, J., and Ma, H. (2019) Protective Effects of the Ginseng Saponin Rh1 Pretreatment to Myocardial Ischemia-reperfusion Injury in Rats. Journal of Liaoning University of TCM. 1: 42-45.
[32] Sun, J. (2013) Research on active ingredients of Chinese medicine and molecular mechanisms based on the myocardial protective effec. [Doctor's Thesis]. [Beijing (China)]: Chinese Academy of Medical Sciences & Peking Union Medical College.
[33] Sun, J., Sun, G., Meng, X., Wang, H., Wang, M., Qin, M., Ma, B., Luo, Y., Yu, Y., Chen, R., Ai, Q., and Sun, X. (2013) Ginsenoside RK3 Prevents Hypoxia-Reoxygenation Induced Apoptosis in H9c2 Cardiomyocytes via AKT and MAPK Pathway. Evid Based Complement Alternat Med. 2013: 690190. doi: 10.1155/2013/690190.
[34] Huang, Q., Su, H., Qi, B., Wang, Y., Yan, K., Wang, X., Li, X., and Zhao, D. (2021) A SIRT1 Activator, Ginsenoside Rc, Promotes Energy Metabolism in Cardiomyocytes and Neurons. J Am Chem Soc. 143: 1416-1427. doi: 10.1021/jacs.0c10836.
[35] Wang, Z. (2008) Protective effects of ginsenoside Rb1 on myocardial ischemia-reperfusion injury and its mechanisms. [Doctor's Thesis]. [Guangzhou (China)]: Sun Yat-sen University.
[36] Liu, C., Wu, S., and Ye, G. (2012) Mechanism of Ginsenoside Rb1 Against Myocardial Apoptosis during Ischemia-reperfusion Injury in Diabetic Rats. JETCM. 21: 1080-1081.
[37] Wu, Y., Xia, Z.Y., Dou, J., Zhang, L., Xu, J.J., Zhao, B., Lei, S., and Liu, H.M. (2011) Protective effect of ginsenoside Rb1 against myocardial ischemia/reperfusion injury in streptozotocin-induced diabetic rats. Mol Biol Rep. 38: 4327-4335. doi: 10.1007/s11033-010-0558-4.
[38] Ma, L., Liu, H., Xie, Z., Yang, S., Xu, W., Hou, J., and Yu, B. (2014) Ginsenoside Rb3 protects cardiomyocytes against ischemia-reperfusion injury via the inhibition of JNK-mediated NF-κB pathway: a mouse cardiomyocyte model. PLoS One. 9: e103628. doi: 10.1371/journal.pone.0103628.
[39] Feng, R., Liu, J., Wang, Z., Zhang, J., Cates, C., Rousselle, T., Meng, Q., and Li, J. (2017) The structure-activity relationship of ginsenosides on hypoxia-reoxygenation induced apoptosis of cardiomyocytes. Biochem Biophys Res Commun. 494: 556-568. doi: 10.1016/j.bbrc.2017.10.056.
[40] Cao, X. (2004) Experimental studies on protective effects of ginsenoside Re on myocardium of ischemia-reperfusion injury. [Doctor's Thesis]. [Jilin (China)]: Jilin University.

[41] Fan, H., Xiaoling, S., Yaliu, S., Mingming, L., Xiansheng, M., and Li, F. (2015) Tissue distribution of Ginsenoside Ｒg3 and its metabolites in the body of rats. Pharmacology and Clinics of Chinese Materia Medica. 31: 16-19.
[42] Wang, Y., and Zhang, Y. (2016) The effect and mechanism of ginsenoside Rb1 in improving myocardial ischemia/reperfusion injury via P38MAPK pathway. World Latest Medicine Information. 16: 89-90.
[43] Li, G., Qian, W., and Zhao, C. (2016) Analyzing the anti-ischemia-reperfusion injury effects of ginsenoside Rb1 mediated through the inhibition of p38α MAPK. Can J Physiol Pharmacol. 94: 97-103. doi: 10.1139/cjpp-2014-0164.
[44] Fu, X., Zhang, Q., Luo, R., and Wang, P. (2020) Protective effect of ginsenoside Rh3 on myocardial ischemia-reperfusion injury in rats by regulation of p38 MAPK/caspase-3 signaling pathway. Int J Clin Exp Med. 13: 3212-3218.
[45] Cao, L., Gao, Y., Zhu, J., Zhang, J., Dong, M., and Mao, Y. (2020) Protective action of the ginsenoside Rh3 in a rat myocardial ischemia-reperfusion injury model by inhibition of apoptosis induced via p38 mitogen-activated protein kinase/caspase-3 signaling. J Int Med Res. 48: 300060520969090. doi: 10.1177/0300060520969090.
[46] Li, J., Yang, Y.L., Li, L.Z., Zhang, L., Liu, Q., Liu, K., Li, P., Liu, B., and Qi, L.W. (2017) Succinate accumulation impairs cardiac pyruvate dehydrogenase activity through GRP91-dependent and independent signaling pathways: Therapeutic effects of ginsenoside Rb1. Biochim Biophys Acta Mol Basis Dis. 1863: 2835-2847. doi: 10.1016/j.bbadis.2017.07.017.
[47] Yan, X., Liu, J., Wu, H., Liu, Y., Zheng, S., Zhang, C., and Yang, C. (2016) Impact of miR-208 and its Target Gene Nemo-Like Kinase on the Protective Effect of Ginsenoside Rb1 in Hypoxia/Ischemia Injuried Cardiomyocytes. Cell Physiol Biochem. 39: 1187-1195. doi: 10.1159/000447825.
[48] Yan, X., Xue, J., Wu, H., Wang, S., Liu, Y., Zheng, S., Zhang, C., and Yang, C. (2015) Ginsenoside-Rb1 Protects Hypoxic- and Ischemic-Damaged Cardiomyocytes by Regulating Expression of miRNAs. Evid Based Complement Alternat Med. 2015: 171306. doi: 10.1155/2015/171306.
[49] Bao, J., Zhang, X., Lu, X., and Rao, M. (2010) Effect of Panax notoginsenoside Rb1 and Rg1 on cell apoptosis of hypoxia-reoxygenation injury in angiotensin II induced hypertrophied neonatal rat myocytes. Gansu Medical Journal. 29: 121-124.
[50] Guan, L., Li, W., and Liu, Z. (2002) Effect of Ginsenoside-Rb1 on Cardiomyocyte Apoptosis after Ischemia and Reperfusion in Rats. Journal of Huazhou University of Science and Technology. 22: 212-215.
[51] Zhang, W., Zhang, W., and Zhong, G. (2002) Insulin resistance model induced by dexamethasone in rats. Chin J Appl Physiol. 18: 99-100.

[52] Lu, X. (2009) Studies on influence of ginsenoside-Re on myocardium ischemia-reperfusion injury and apoptosis pathway of mitochondrial. [Master's Thesis]. [Jilin (China)]: Jinlin University.
[53] Gao, Y., Yang, J., Wang, Y., and Wang, Q. (2011) Effect of Ginsenoside Re on Rat Myocardial Ischemia-Reperfusion Apoptosis and Caspase-3. Journal of Liaoning University of TCM. 13: 123-124.
[54] Zhang, Z.L., Fan, Y., and Liu, M.L. (2012) Ginsenoside Rg1 inhibits autophagy in H9c2 cardiomyocytes exposed to hypoxia/reoxygenation. Mol Cell Biochem. 365: 243-250. doi: 10.1007/s11010-012-1265-3.
[55] Xin, G. (2020) Study on the mechanism of Shuangshen Ningxin apsule regulating FUNDC1 mediated mitochondrial autophagy to protect myocardial ischemia-reperfusion injury. [Master's Thesis]. [Beijing (China)]: China Academy of Chinese Medical Sciences.
[56] Zhou, C. (2009) The protective effect of Rg2 on hypoxic myocardial cells. [Master's Thesis]. [Qingdao (China)]: Qingdao University.
[57] Tian, J., Zheng, S., Guo, W., Ye, J., and Li, L. (2004) Ginsenoside Rg2 protection against apoptosis in ischemia and reperfusion rat myocardium. CHinese Pharmacological Bulletin. 20: 480.
[58] Wang, J., Cui, Y., Wang, J., Li, H., Dai, J., and Ma, H. (2016) Effect of ginsenoside Rh3 pretreatment on expression of myocardial sarcoplasmic reticulum Ca^{2+}-ATP in rats following I/R injury. Chin J Geriatr Heart Brain Vessel Dis. 18: 1077-1081.
[59] Wang, J., Cui, Y., Wang, J., Li, H., Dai, J., and Ma, H. (2017b) Protective effects of the ginseng saponin Rh3 pretreatment to caspase-3 of myocardial ischemia-reperfusion injury in rats. Journal of Changchun University of Chinese Medicine. 33: 13-15.
